# Assessment of knowledge and practice of community pharmacy personnel on diabetes mellitus management in Kathmandu district: a cross sectional descriptive study

**DOI:** 10.1186/s40200-015-0205-7

**Published:** 2015-09-21

**Authors:** M. Shrestha, R. Maharjan, A. Prajapati, S. Ghimire, N. Shrestha, A. Banstola

**Affiliations:** Department of Pharmacy, Valley College of Technical Sciences, Kathmandu, Nepal; Department of Public Health, Valley College of Technical Sciences, Kathmandu, Nepal

**Keywords:** Community pharmacy in Nepal, Diabetes mellitus, Pharmacy practice

## Abstract

**Background:**

Pharmacists are the most reachable healthcare professionals to many chronically ill patients. It has been found that pharmacists see patients with diabetes up to five times more often than any other healthcare provider. Therefore, to provide quality health care to patients it is important that they have appropriate knowledge and practice on diabetes mellitus management. Thus, this study was conducted to assess the knowledge and practice of diabetes mellitus management among community pharmacy personnel involved in retail community pharmacies of Kathmandu.

**Methods:**

Three hundred and fifteen community pharmacies, selected by systematic random sampling were surveyed by using pre-validated self-administered questionnaires. The first set of questionnaire evaluated the community pharmacy personnel’s diabetes knowledge based on a pre-validated 20-item questionnaire. The second set of questionnaire documented about the practice of community pharmacy personnel on diabetes mellitus management which contained 22 questions. Data was entered in EPI Data and analyzed by using SPSS version 20.

**Results:**

This survey demonstrated that 76.5 % respondents had poor knowledge and 86.4 % had negative practice on diabetes mellitus (DM) management. Only 26.2 % respondents had good knowledge as well as good practice. 31.4 % of respondents had poor knowledge as well as poor practice on DM management.

**Conclusions:**

Laws and regulations regarding community pharmacy personnel need to be implemented. There should be more advanced and experiment based training. Additionally, the provision for further education curriculum in pharmacy education should be implemented which should intensively include disease and proper management. Guidelines covering diabetes care should be distributed and implemented throughout community pharmacies.

## Background

Diabetes mellitus is a chronic disease that constitutes a major public health problem. The worldwide prevalence of DM has risen tremendously over the past two decades [1]. It has been projected that the number of individuals with DM will continue to increase in the near future [2]. The prevalence of diabetes among adults (20–79) years Nepal is 4.2 % [3]. In Kathmandu, Nepal, diabetic prevalence ratio in male is 27.1 % and in female is 24.8 % [4].

The proper control of the illness is dependent on the patient’s adherence to medications, life style modifications, frequent monitoring of blood glucose, etc. and can be influenced by proper education and counselling of the patient. Pharmacists, being one of the indispensable members of the health care team, have an immense responsibility for counselling patients. Pharmacist consultations provided to patients with diabetes can minimize total healthcare costs in a health maintenance organization [5]. Community pharmacist’s intervention on improving knowledge and glycemic control has shown better progress in recovery of diabetics. Continuous counselling and monitoring plays an important role in the improvement of glycemic control [6]. Intervention by community pharmacist has a beneficial effect on the clinical management of type II DM [7]. Assessing the knowledge and practice of community pharmacy personnel can help to design appropriate targeted educational training for diabetic patients’ benefit.

In Nepal, community pharmacy is the first place that people visit for consultation regarding health problems, getting medications and refilling their prescription. Modern pharmacy education in Nepal is relatively new. In the past, community pharmacies were managed by people with basic orientation training in drug dispensing and basic pharmacology. Today though the Drug Act of Nepal 1978 mandates the presence of pharmacists, with a degree of bachelor in pharmacy or pharmacy assistants with a degree of diploma in pharmacy in community pharmacy, people from diverse educational background with orientation training are still working as community pharmacy personnel [8]. Due to poor medical services, the public awareness regarding DM in Nepal is below the satisfactory level [9]. Because of inadequate public awareness, diabetic patients have insufficient level of knowledge, attitude and practice regarding their disease [10]. This can be ameliorated if community pharmacy personnel have adequate knowledge and practice regarding the disease to advise the patient. There are virtually no epidemiological studies from Kathmandu assessing the level of knowledge and the practice of community pharmacy personnel regarding Diabetes Mellitus (DM). Hence, this research seeks high attention to evaluate whether the community pharmacy personnel in Kathmandu have the sufficient knowledge about DM and to assess their practice for the management of this disease and its complications.

## Methods

### Sample

This study used a cross-sectional descriptive design. The study population was 1762 retail community pharmacies of Kathmandu registered in Department of Drug Administration (DDA), Nepal [11].

The sample size was calculated by using stat calculator in EPI-INFO version 7; based on statistical formula, sample size (ss) = z^2^pq/d^2^; taking z = 1.96 at 95 %, p (prevalence) = 50 %, q = 1 − p and d (allowable error) = 5 %. Further, sample size for finite population was calculated by using formula, *n* = ss/(1 + (ss − 1)/p) and finite population size (p) 1762.

Thus, the population based sample size at 95 % confidence interval and 50 % prevalence was 315.

Sampling frame was developed from the list of retail pharmacy, in alphabetical order, obtained from DDA, Nepal. Systemic random sampling was used to select 315 community pharmacies. Random number was generated using the software Decision Analyst, STATS^TM^2.0. If the selected pharmacies were closed during the period of data collection, they were revisited next day. If pharmacy in the sample list was not available, shifted from the location mentioned in DDA list, not operating anymore as informed by the local residents, alternative pharmacy, within the sampling frame, in nearby location was chosen.

### Ethics statement

Ethical approval was taken from Ethical Review Board (ERB) at Nepal Health Research Council. Informed verbal consent was taken from the respondents prior to the final data collection. The respondents took voluntary participation and identity of respondents was kept confidential.

### Instrument

Self-administered questionnaire was used to acheive aforesaid objectives. Twenty item questionnaire on knowledge of diabetes was adapted from pre validated Diabetes Knowledge Test (DKT) developed by the Michigan Diabetes Research Training Centre (MDRTC). Twenty-two item questionnaire on practice on diabetes management was based on tools developed by Qatar University. Permission was taken from the respective authors to use the tools.

The questionnaires in English language were translated into Nepali by the researchers. Nepali tools were further back translated to English by a person other than the researchers who had good command in both English and Nepali language to check the correctness of the Nepali translation. The questionnaires were slightly modified to match with Nepali context. Nepali questionnaires were pretested among 5 % of the final sample size, i.e. among 16 community pharmacy personnel in Bhaktapur district. The pre-tested samples were not included in final data of the study. Then final version of the questionnaire was developed after pre-test.

### Methodology

About 15 to 20 minutes time was taken by each respondent, i.e. one pharmacy personnel from each selected community pharmacy, to fill up the questionnaire. Since they filled up the questionnaire in the presence of researcher, there was less chance of bias during answering the questions. The respondents were encouraged to ask questions in case of confusion regarding questionnaires. The questionnaire form was checked immediately after it had been filled up by the respondent to find if any question had been unattended.

Questions on knowledge had only one “correct” option and rest were “wrong” answers. One point was given for each correct answer and the knowledge score was calculated by adding up the points for each questions. The total knowledge score was 20. For respondents’ convenience, “Don’t know” option was also included in each question but considered as incorrect answer.

Questions on practice had options “never”, “rarely”, “frequently”, “always”. The “always” option was considered as good practice and other options were considered as negative practice. One score was given to each “always” option and the practice score was calculated by adding the points for each “always” answer. The total knowledge score was classified into “good knowledge” for score ranging (17–20), “moderate knowledge” for (13–16) and poor knowledge” for (0–12) and practice was classified into “positive practice” for score ranging (16–22) and “negative practice” for (0–15) based on the classification by Al-maskari et.al by taking the percentage for each range [12]. In order to meet the objective of finding out the gap between knowledge and practice, mean and mean deviation for knowledge and practice was calculated. Plotting the mean deviation for knowledge against mean deviation for practice resulted in four quadrants with following specific characteristics:

1st quadrant showed the proportion of respondents who had good knowledge and good practice, 2nd quadrant indicated the proportion of respondents who had good practice but poor knowledge, 3rd quadrant resembled to the proportion of respondents who had poor knowledge and poor practice and the 4th quadrant showed the proportion of respondents who had good knowledge but poor practice regarding DM management.

### Data processing and analysis

The data was entered into standardized data entry mask, developed in EPI Data, on the same day of data collection. To ensure the accuracy and quality of data entry, 10 % of total sample was randomly selected by generating random number using Decision Analyst Stat 2.0 software. The data in selected questionnaire were then manually tallied with computer data entry. The data was then analysed using the SPSS version 20 computer software package. A descriptive data analysis was performed to access the respondents’ socio-demographic characters.

## Results

### Socio-demographic characteristics

Seventy-three percent of respondents were male (95 % C.I. 68, 78) and majority of the respondents (35.2 %) belonged to the age group 20–29. Only 33 % of the respondents were either pharmacists or pharmacy assistants (Table 1).

**Table 1:** Socio-demographic characteristics

Category	Frequency	Percent
Sex		
Female	86	27.3
Male	229	72.7
Age		
19 or less	9	2.9
20–29	111	35.2
30–39	100	31.8
40–49	60	19.0
50–59	27	8.6
60 and above	8	2.5
Educational status		
Pharmacists and pharmacy assistants	104	33
Others with pharmacy related training	180	57.2
Others without pharmacy related training	31	9.8
Total	315	100.0

### Knowledge and practice of community pharmacy personnel regarding DM management

The mean score of knowledge was 10.67 (95 % C.I. 10.37, 10.97) and the mean score of practice was 6.78 (95 % C.I. 6.0, 7.5). Majority of the respondents i.e. 76.5 % had scored between 0 and 12, which corresponds to poor knowledge on DM management and only 1.9 % of the respondents had good knowledge. Sixty-eight respondents (21.6 %) had moderate level of knowledge i.e. they obtained scores ranging from 13 to 16. Only 13.6 % of the respondents had a positive practice on DM management. A massive number of the respondent had negative practice i.e. 86.4 % as per definition set in the study (Table 2).

**Table 2:** Comparison of Knowledge and Practice regarding DM management

Score category	Percent %
Knowledge	(*N* = 315)
Good knowledge	6 (1.9 %)
Moderate knowledge	68 (21.6 %)
Poor knowledge	241 (76.5 %)
Practice	(*N* = 309)
Positive practice	42 (13.6 %)
Negative practice	267 (86.4 %)
Total	100 %

### Frequency of diabetes care services

Counselling regarding the use and interpretation of result of glucose meters was never practiced by 34 % and 39.8 % of the respondents respectively. Likewise 34 % of respondents never practiced counselling about the cautions of use of other herbal. In comparison to other activities, promotion of smoking cessation and describing the appropriate time of oral anti-diabetic drug administration was done more frequently (Table 3).

**Table 3:** Frequency of diabetes care services

Diabetes care services	Never		Rarely		Often		Always	
	Frequency	%	Frequency	%	Frequency	%	Frequency	%
Counselling on the appropriate handling of insulin (*N* = 309)	93	30.1	79	25.6	61	19.7	76	24.6
Counselling on the appropriate storage of insulin (*N* = 309)	81	26.2	60	19.4	65	21.0	103	33.3
Counselling on appropriate insulin administration (*N* = 309)	105	34.0	58	18.8	65	21.0	81	26.2
Counselling on method of using blood glucose meters? (*N* = 309)	123	39.8	70	22.7	53	17.2	63	20.4
Counselling on result interpretation of glucose meters (*N* = 310)	129	41.6	64	20.6	50	16.1	67	21.6
Counselling on the causes of hypoglycaemia (*N* = 309)	79	25.6	91	29.4	78	25.2	61	19.7
Counselling about symptoms of hypoglycaemia (*N* = 309)	78	25.2	99	32.0	78	25.2	54	17.5
Counselling about treatment of hypoglycaemia (*N* = 310)	69	22.3	94	30.3	84	27.1	63	20.3
Counselling on matter of other illness (*N* = 310)	38	12.3	99	31.9	89	28.7	84	27.1
Counselling on matters of stress and tension (*N* = 310)	50	16.1	96	31.0	77	24.8	87	28.1
Counselling on the cautions of OTC drugs as they relate to diabetes management (*N* = 309)	32	10.4	75	24.3	76	24.6	126	40.8
Counselling on the cautions of herbal drugs as they relate to diabetes management? (*N* = 310)	119	38.4	79	25.5	45	14.5	67	21.6
Provide education on regular screening for nephropathy (*N* = 309)	33	10.7	94	30.4	96	31.1	86	27.8
Provide education on regular screening for retinopathy? (*N* = 309)	20	6.5	111	35.9	88	28.5	90	29.1
Provide education on regular screening for neuropathy? (*N* = 309)	59	19.1	104	33.7	78	25.2	68	22.0
Counselling on good foot care techniques? (*N* = 310)	57	18.4	112	36.1	61	19.7	80	25.8
Stress the importance of weight control in diabetes management where applicable? (*N* = 310)	23	7.4	79	25.5	80	25.8	128	41.3
Stress the importance of diet in diabetes management (*N* = 309)	18	5.8	72	23.3	87	28.2	132	42.7
Stress the importance of regular exercise in diabetes management (*N* = 309)	15	4.9	66	21.4	92	29.8	136	44.0
Promote smoking cessation (where applicable) (*N* = 310)	17	5.5	56	18.1	79	25.5	158	51.0
Describe the appropriate time to administer each oral anti-diabetic drug (*N* = 309)	25	8.1	48	15.5	78	25.2	158	51.1
Counselling regarding missed oral anti-diabetic dose (*N* = 310)	40	12.9	71	22.9	72	23.2	127	41.0

### Gap of knowledge and practice of respondents

**Fig. 1:**
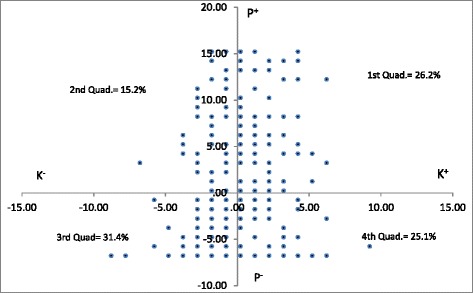
Gap of knowledge and Practice of Community pharmacy personnel; The plot shows the comparison of knowledge and practice of the respondents, *X axis* shows the knowledge of respondents regarding DM and *Y axis* shows their respective practice on DM management

In Fig. 1, Quadrant 1 shows that 26.2 % of the respondents had good knowledge about diabetes mellitus as well as good practice on diabetes mellitus management. Quadrant 2 shows that 15.2 % of the respondents had good practice but poor knowledge on DM management. Quadrant 3 shows that 31.4 % of the respondents had poor knowledge as well as poor practice on DM management. Quadrant 4 shows that 25.1 % of the respondents had poor practice but good knowledge on DM management.

## Discussion

Intensive diabetes education and care supervision can progress patient outcomes, glycemic control and improve standard of life in patients [13]. The current study is first of its kind to access the knowledge and practice of community pharmacy personnel regarding DM management in Nepal which will contribute to take necessary steps to improve the quality of life of diabetic patients.

There were several important findings of the study. Surprisingly, 9.8 % of pharmacies were run by a single staff who had not studied health sciences at all. Considering the lack of essential knowledge and skills of service providers, such finding clearly suggests that effective quality care of patients cannot be safeguarded. Majority of the respondents had low level of knowledge on diabetes which is in line with the findings of study conducted in Libya and Qatar. The study conducted in Qatar showed that 26 % of the respondents scored less than 60 % in diabetes knowledge test [14]. The general diabetes knowledge of community pharmacists in Libya was found worse than their knowledge regarding insulin therapy [15]. The reasons of poor knowledge in our study could be lack of continuing education program. Conducting continuing education program on pharmacists for enhancing the ability to perform pharmaceutical care for diabetic care has shown to increase the participant’s knowledge [16]. The low level of knowledge could be the reason for low caliber of practice in DM management which has been depicted by the low mean score of practice in this study. Another reason for low level of knowledge and practice among the respondents in this study may be due to the fact that in Nepal, community pharmacy personnel come from diverse educational background, and thus may not have the intensive knowledge and practice skill required for their profession. The study conducted in North-East region of UK also suggested that the practice of community pharmacist on type 2 DM was not within the set standards and objectives [17]. Lack of time, inadequate human resources, lack of therapeutic knowledge, lack of confidence and lack of provision of counseling service are the reasons for the practice to be below satisfactory level [18]. However, comparatively high rate of counseling on lifestyle found in our study was in contrast with the result of Scottish, American and Norwegian studies [17, 19]. Lack of reimbursement, constraints while working, physical design of the pharmacy, and lack of training were believed to be the cause in those studies [19]. In context of Nepal, there are no appropriate guidelines for proper counseling of diabetic patients for the disease management.

In this study, 26.2 % of the respondents who had good knowledge about diabetes mellitus and good practice on diabetes mellitus management should be encouraged to continue with their good practice by offering various incentives. The 25.1 % of the respondents who had good knowledge but poor practice on DM management should be targeted for behaviour change programme. Additionally, despite the good knowledge, this group has poor practice, thus, the barriers to good practice should be identified and addressed. And 15.2 % of the respondents who have poor knowledge but good practice on DM management should be targeted for trainings to enhance their knowledge whereas 31.4 % of the respondents who had both poor knowledge as well as poor practice on DM management should be the main focus of any intervention aiming to enhance the knowledge and practice on DM management. The knowledge of this group on DM can be enhanced by appropriate trainings. Similarly, behaviour change programme should be provided to enhance their practice on DM management. The only limitation of this study was un-traceability of sample location as there was no recent updated DDA list.

## Conclusion

The low level of knowledge and negative practice of DM management among community pharmacy personnel as depicted in this study recommends that there should be more advanced, experimental based training and provision for further education curriculum in pharmacy education, which should intensively include disease and its proper management. Guidelines covering diabetes care should be implemented and distributed throughout community pharmacies. Regular monitoring and inspections regarding the implementation of laws are of utmost importance in perking up the development of diabetes care in community pharmacies.
